# Pleiotropic Functions for Transcription Factor Zscan10

**DOI:** 10.1371/journal.pone.0104568

**Published:** 2014-08-11

**Authors:** Petra Kraus, Sivakamasundari V, Hong Bing Yu, Xing Xing, Siew Lan Lim, Thure Adler, Juan Antonio Aguilar Pimentel, Lore Becker, Alexander Bohla, Lillian Garrett, Wolfgang Hans, Sabine M. Hölter, Eva Janas, Kristin Moreth, Cornelia Prehn, Oliver Puk, Birgit Rathkolb, Jan Rozman, Jerzy Adamski, Raffi Bekeredjian, Dirk H. Busch, Jochen Graw, Martin Klingenspor, Thomas Klopstock, Frauke Neff, Markus Ollert, Tobias Stoeger, Ali Önder Yildrim, Oliver Eickelberg, Eckhard Wolf, Wolfgang Wurst, Helmut Fuchs, Valérie Gailus-Durner, Martin Hrabě de Angelis, Thomas Lufkin, Lawrence W. Stanton

**Affiliations:** 1 Stem Cell and Developmental Biology, Genome Institute of Singapore, Singapore, Singapore; 2 Department of Biology, Clarkson University, Potsdam, New York, United States of America; 3 German Mouse Clinic, Helmholtz Zentrum München, German Research Center for Environmental Health, Neuherberg, Germany; 4 Institute of Experimental Genetics, Helmholtz Zentrum München, German Research Center for Environmental Health, Neuherberg, Germany; 5 Institute of Developmental Genetics, Helmholtz Zentrum München, German Research Center for Environmental Health, Neuherberg, Germany; 6 Institute of Pathology, Helmholtz Zentrum München, German Research Center for Environmental Health, Neuherberg, Germany; 7 Chair for Molecular Animal Breeding and Biotechnology, Gene Center, Ludwig-Maximilians-Universität München, Munich, Germany; 8 Department of Medicine III, Division of Cardiology, University of Heidelberg, Heidelberg, Germany; 9 Institute of Medical Microbiology, Immunology, and Hygiene, Technische Universität München, Munich, Germany; 10 Molecular Nutritional Medicine, Else Kröner-Fresenius Center, Technische Universität München, Freising-Weihenstephan, Germany; 11 Department of Neurology, Friedrich-Baur-Institut, Ludwig-Maximilians-Universität München, Munich, Germany; 12 Klinikum rechts der Isar der Technischen Universität München, Klinik und Poliklinik für Dermatologie und Allergologie am Biederstein, Munich, Germany; 13 Comprehensive Pneumology Center, Institute of Lung Biology and Disease, Helmholtz Zentrum München, German Research Center for Environmental Health, Neuherberg, Germany; 14 Chair of Experimental Genetics, Center of Life and Food Sciences Weihenstephan, Technische Universität München, Freising-Weihenstephan, Germany; 15 Member of German Center for Diabetes Research, Neuherberg, Germany; 16 Chair of Developmental Biology, Technische Universität München, Freising-Weihenstephan, Germany; 17 Max Planck Institute of Psychiatry, Munich, Germany; 18 Deutsches Institut für Neurodegenerative Erkrankungen Site Munich, Munich, Germany; 19 Munich Cluster for Systems Neurology, Munich, Germany; National Cancer Institute, United States of America

## Abstract

The transcription factor Zscan10 had been attributed a role as a pluripotency factor in embryonic stem cells based on its interaction with Oct4 and Sox2 in *in vitro* assays. Here we suggest a potential role of Zscan10 in controlling progenitor cell populations *in vivo*. Mice homozygous for a *Zscan10* mutation exhibit reduced weight, mild hypoplasia in the spleen, heart and long bones and phenocopy an eye malformation previously described for *Sox2* hypomorphs. Phenotypic abnormalities are supported by the nature of *Zscan10* expression in midgestation embryos and adults suggesting a role for Zscan10 in either maintaining progenitor cell subpopulation or impacting on fate choice decisions thereof.

## Introduction

Zscan10 (previously known as Zfp206) is a 14 zinc finger transcription factor (ZF-TF) of the C_2_H_2_ ZF-TF subfamily with a N-terminal SCAN domain [1], [2] located on mouse chromosome 17. It was discovered during an array screen in mouse [3] and a transcriptome characterization of human embryonic stem cells [4]. *Zscan10* has at least six known alternative splice forms, is expressed in undifferentiated human and mouse embryonic stem cells (ESC) [3], [4], the inner cell mass (ICM) of blastocysts [5] and down regulated upon differentiation [3], [6]. Zscan10 is considered to maintain ESC pluripotency by interacting with the established pluripotency markers Sox2 and Oct4 [7]. More than 3000 binding sites were identified for Zscan10 in a genome wide screen of which 183 were commonly targeted by this trio [1]; suggesting a direct or indirect role of Zscan10 in controlling many developmental processes by activating or suppressing other transcription factors. Post-implantation expression of the *Zscan10* isoform 2 has been reported by whole mount RNA *in situ* hybridization (WISH) in the ecto- and mesoderm but not the endoderm of embryonic day 7.5 (E7.5) mouse embryos. Expression remained in the otic vesicle, the branchial arches and the hindgut between E9.0–9.5 and became barely detectable at E10.5 [3], while WISH using a probe based on the last exon of *Zscan10* indicated expression in the ectoderm and mesoderm but not endoderm of E7.5 embryos and low level expression in all germ layers from E9.5 to E12.5. Northern blot analysis performed with a 60 bp oligonucleotide located in the last exon of *Zscan10* revealed strongest expression in embryos at E4.5–E5.5 and E10.5–E13.5, while in the adult expression was limited to the testis [6]. Subsequently qPCR analysis indicated low level expression in brain and liver. Array data from Zhang *et al*. indicated upregulation of *Zscan10* expression in E9.5 embryos as well as lymph nodes, the spinal cord, testis and epididymis as well as femur and calvarial bones [3]. While the last exon is present in the most abundant isoforms of *Zscan10*, only exon 4 is shared amongst all six known alternative splice variants [3].

During post-fertilization pre-implantation stages in mouse, a totipotent zygote develops into a pluripotent blastocyst by E3.5 with only the cells of the ICM retaining the pluripotent state. Pluripotent ESC can be derived from the ICM of blastocysts [8], [9]. Upon injection of pluripotent ESC into 2C–8C stage pre-implantation embryos [10] or blastocysts [11] chimeric organisms can be generated. The degree of chimerism is determined by the degree of pluripotency of any given injected ESC and its ability to outcompete cells of the host organism to achieve contribution to the chimeric organism and from an evolutionary point of view, most importantly its germ line [10]. Upon undergoing differentiation, a pluripotent stem cell can divide into a terminally differentiated cell or become a more specialized progenitor cell with multi- or unipotent potential [12].

Here we propose a possible role for Zscan10 in the maintenance of multipotent progenitor cells based on the pleiotropic phenotype revealed by an exhaustive screen of *Zscan10* homozygous mutant mice derived from clone #285B6 (CMMR) of a gene trap located 5′ to the ATG start codon of Zscan10, which should render null all known protein isoforms of Zscan10, yet allow residual 3′ RNA expression to persist. Typically affected organs did retain residual *Zscan10* RNA expression in distinct cells during midgestation development and even in the adult and were generally determined to be hypoplastic in the *Zscan10* homozygous mutants.

## Materials and Methods

### Generation of homozygous mutant *Zscan10* gene trap mice

*Zscan10* mutant mice were generated by injecting the R1 mouse ESC clone #285B6, obtained from the Canadian Mouse Mutant Repository (CMMR) (Toronto, Canada), into C57BL/6 2C–8C stage embryos as described in Kraus *et al*. [10], achieving germline transmission and generating a stable line that was backcrossed for more than three generations onto a C57BL/6 background. According to CMMR clone #285B6 resulted from a gene trap insertion of the pUPA vector prior to the translation start codon of *Zscan10* as confirmed by 3′RACE performed by CMMR. For a schematic of the gene trap allele see Figure S1. As recommended by CMMR, heterozygous mice were mated with the *ZP3*-Cre deleter strain [13] to remove a floxed IRESx3 sequence inherent to the pUPA gene trap vector used by CMMR. Genotyping was performed by PCR using the AccuPrime Taq DNA polymerase system on 50 ng of genomic DNA isolated from tail tips for 30 cycles at 94°C for 30 s/58°C for 30 s/68°C for 60 s with primers for the mutant (285B6-mut-F: 5′-CGATGATCTCGTCGTGACCC-3′/ 285B6-mut-R: 5′-CGGGTCAAATTACGAGGTGCT-3′) and wild type allele (285B6-wt-F: 5′-CTGGCAAGTCCTCACCTAGAAATT-3′/ 285B6-wt-R: 5′-CTGCAGCAGATGCTCGTGAGTT-3′), generating a 300 bp wild type allele and a 1100 bp and 500 bp mutant allele before and after Cre recombination, respectively.

### Ethics statement and animal housing

All animal procedures were performed according to the Singapore A*STAR Biopolis Biological Resource Center (BRC) Institutional Animal Care and Use Committee (IACUC) guidelines which are set by the National Advisory Committee for Laboratory Animal Research (NACLAR). The IACUC protocols employed were reviewed and approved by the aforementioned committee before any animal procedures were undertaken for this study described here (IACUC Protocol No: 110689 and 110648). The mouse strains used in this study were housed, maintained and provided by the A*STAR Biopolis Biological Resource Center (BRC) (Singapore) & the German Mouse Clinic (GMC) (Neuherberg, Germany) and maintained in IVC cages according to BRC and GMC housing conditions and Singaporean and German laws. All tests performed at the GMC and described here were approved for the ethical treatment of animals by the responsible authority of the Regierung von Oberbayern (Government of Upper Bavaria). The lines described here will be made available to the research community upon acceptance of the manuscript.

### RNA *in situ* hybridization and Western blot

RNA *in situ* hybridization on 10 um paraffin sections (SISH) was carried out, essentially as described [14], with the exception that the probes were generated by PCR amplification of wild type mouse cDNA derived from embryonic E12.5 and R1 ESC, as described [15], [16]. Several *Zscan10* probes have been used for SISH, emphasized here is the 270 bp probe template that spans exons 3 to 5 of the *Zscan10* cDNA, precisely from base pair position 667 to 937 of the reference sequence NM_001033425 and the 1355 bp probe template spanning base positions 433 to 1788. PCR was carried out following standard PCR procedures using GoTaq (Promega) at 1.0 mM MgCl_2_ on a Nexus Eppendorf thermocycler with a 40 s annealing step at 65°C and 2.0 mM MgCl_2_ at 60°C, respectively, for 35 cycles with primers including the sequence for the T3 and T7 RNA polymerase promoters, to generate a DIG labeled sense and antisense probe, respectively, using the DIG RNA labeling mix (Roche):

Zfp206_T3: 5'-CGCGCGTTAATTGGGAGTGATTTCCCCCTCAGAAGAGATTCCAGCCC-3', Zfp206_T7: 5'-GCGCGCGTAATACGACTCACTATAGGGCGCCAAGCTCTCTTCTCTGAGGT-3', Zscan10_433_T3: 5′-CGCGCGCAATTAACCCTCACTAAAGGGCTGGAGCAGTTCCTGAGTGTC-3′, Zscan10_1788_T7:5′-GCGCGCGTAATACGACTCACTATAGGGCTAGTTGCTCGCTTTGTCGGAA-3′. Likewise a ∼1 kb probe for *Sox2* was generated using the primers: T7_Sox2_S: 5′- GCGCGCGTAATACGACTCACTATAGGGCTTTGTCCGAGACCGAGAAGC-3′, T3_Sox2_AS: 5′- CGCGCGCAATTAACCCTCACTAAAGGGGAAGCGCCTAACGTACCACT-3′. The ORF *Hes5* probe was a generous gift from Urban Lendahl. The probe template for the pUPA reporter gene EGFP was amplified using the primers EGFP_T7: 5′-CGCGCGCAATTAACCCTCACTAAAGGGCCTACGGCGTGCAGTGCTTCAGC-3′ and EGFP_T3: 5:-CGCGCGGTAATACGACTCACTATAGGGCCGGCGAGCTGCACGCTGCCGTCC-3′.

Western blot was essentially performed as described [6] with a Zscan10 specific antibody at 1∶3000 and aRB-HRP 1∶10000 as control (see Figure S5). For comparison wild type (+/+), heterozygous (+/−) and homozygous (−/−) ESC were derived from blastocysts of *Zscan10* ^+/−^ x *Zscan10* ^+/−^ matings. The homozygous (−/−) line L2 was maintained on gelatinized plates in the absence (GEL) or presence (MEF) of mouse embryonic fibroblast feeder cells all maintained in ES medium+LIF.

### Phenotype screen at GMC

The GMC screen (www.mouseclinic.de) comprises exhaustive, standardized phenotyping in the realms of dysmorphology, cardiovascular health, energy metabolism, clinical chemistry, eye, lung function, molecular phenotyping, behavior, neurology, nociception, immunology, steroid metabolism and pathology. Phenotypic screens at the GMC were performed according to standardized methods [17], [18], [19]. A cohort of each 15 male and 15 female wild type and homozygous mutant adult mice derived from timed heterozygous x heterozygous matings, all born within one week, was subjected to the GMC phenotyping program between the age of 9 to 18 weeks. If not stated otherwise, data generated by the GMC was analyzed using R. Tests for genotype effects were made by using t-test, Wilcoxon rank sum test, linear models, or ANOVA depending on the assumed distribution of the parameter and the questions addressed to the data. A p-value <0.05 has been used as level of significance; a correction for multiple testing has not been performed.

### Dysmorphology and skeletal analysis

Macroscopic examination to identify any morphological abnormalities was performed according to GMC standard operating procedures (SOP). Growth weight and body size was determined. Mice were subjected to morphological observation and hearing abilities were analyzed following the protocol described previously [20]. X-ray imaging of animals was performed in a Faxitron MX-20 Cabinet X-ray system equipped with a DC12 digital camera (Faxitron X-ray, Illinois, USA). The system is run with settings: Voltage 26 kV and automatic exposure control. Bone mineral density was analyzed using pDEXA Sabre X-ray Bone Densiometer (Norland Medical Systems. Inc., Basingstoke, Hamshire, UK) scan speed 20 mm/s, resolution 0.5 mm×1.0 mm, Histogram Averaging Width setting 0.020 g/cm^2^.

### Eye analysis and pathology

Morphological alterations of the eye such as anterior segment abnormalities were assessed by Scheimpflug imaging, posterior segment abnormalities, eye fundus and retina were analyzed by optical coherence tomography. The axial eye length was measured by laser interference biometry [21] and visual properties were studied in tests involving a virtual drum [22]. For microscopic histological analysis all organs (skin, heart, muscle, lung, brain, cerebellum, thymus, spleen, cervical lymph nodes, thyroid, parathyroid, adrenal gland, stomach, intestine, liver, pancreas, kidney, reproductive organs and urinary bladder) were fixed in 4% buffered formalin, embedded in paraffin, sectioned at a thickness of 2 um and subjected to hematoxylin and eosin staining as described before [19].

### Neurological examination and behavioral testing

For the assessment of basic neurological functions, muscle function as well as motor coordination and balance, a modified SHIRPA protocol was applied and grip strength and Rota rod performance were measured as described previously [19]. Behavioral screens to analyze locomotion and anxiety-related behavior as well as sensory motor gaiting were done by open field (OFT), acoustic startle and prepulse inhibition (PPI) testing as described before [23], [24].

### Clinical chemistry, hematology and immunology assessment

Blood samples were collected at 16 weeks of age by retrobulbar puncture under isoflurane anesthesia. Determination of clinical-chemical parameters in heparinized plasma samples was done using an AU480 (Beckman-Coulter, Krefeld, Germany) and reagents kits provided by Beckman-Coulter, as described previously [25]. Hematological analyses were performed with an ABC-animal blood counter (Scil animal care company, Viernheim, Germany) on whole blood samples collected in EDTA-coated tubes. Glucose tolerance was tested by an intraperitoneal glucose tolerance test (IpGTT) performed after overnight food withdrawal using the Accucheck Aviva glucometer (Roche, Penzberg, Germany) for glucose blood level determination, as described previously [26]. Analysis of peripheral blood samples was done by flow cytometry and Bioplex multiplex bead array [17], [19].

### Heart and lung function screen

Awake echo was used for the assessment of cardio-vascular functions [26] while assessment of lung volumes and mechanics was performed by Buxco FinePointeRC and Forced Maneuvers systems as described previously [19].

### Energy and steroid metabolism

Measurement of body mass, body temperature, locomotor activity, O_2_ consumption, CO_2_ production, respiratory exchange ratio and body composition was assessed by indirect calorimetry and qNMR (MiniSpec LF50, Bruker Optics, Ettlingen/Germany). The steroid hormones testosterone, corticosterone and androstendione were analyzed out of 50 ul plasma by liquid chromatography tandem mass spectrometry (LC-MS/MS) according to Rathkolb et al [25].

## Results

### Underrepresentation of homozygous *Zscan10* mutant females at weaning age

While homozygous *Zscan10* mutants were viable and fertile, genotyping offspring (n = 153) from heterozygous x heterozygous matings at weaning age (postnatal day 21) revealed a slight deviation from the expected Mendelian ratio, with the number of homozygous females being 30% lower than that of wild type females (see Table S1). Notably, homozygous mice were fertile and could be maintained as homozygous x homozygous, yet their breeding performance generally dropped after a few litters and the offspring appeared increasingly runted (data not shown). Therefore, heterozygous x heterozygous matings were preferred to maintain the *Zscan10* mutant mouse line. *Zscan10* is known to have several alternative splice forms (see Figure S1). While the integration of the gene trap allele will not prevent residual RNA transcripts from being generated, it is very unlikely that any of the Zscan10 protein isoforms are produced (see Figure S5).

### Decreased body weight, bone mass and bone mineral content in *Zscan10* homozygous mutant females

**Figure 1:**
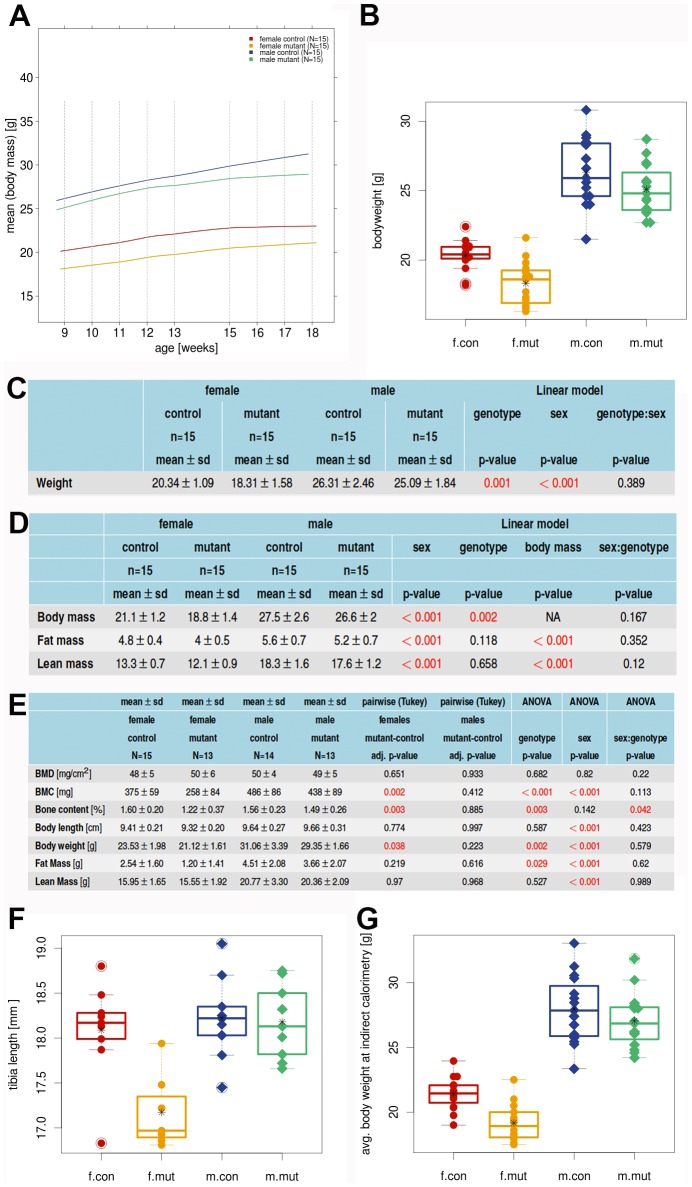
*Zscan10* mutant females show decreased body weight. (A) Body mass time curve of mutant and control cohorts, n = 60. (B) Body weight boxplot with strip chart, split by sex and genotype. Bodyweight is significantly reduced in female mutant mice (p = 0.042). (C) Bodyweight means, standard deviation and p-values calculated by a linear model (D) Body composition analysis by NMR: means, standard deviation and p-values of a linear model. (E) DXA analysis: Group means, standard derivation and ANOVA (Tukey multiple comparisons of means). (F) Tibia length box plot with strip chart. Split by sex and genotype. Tibia length is significantly reduced in female mutant mice. (G) Indirect calorimetry: Average body weight at indirect calorimetry boxplot with strip chart, split by sex and genotype. Points are individual animals; circles represent females (f); diamonds represent males (m) of *Zscan10* homozygous mutants (mut) and wild type littermates (con). Line within the boxplot represents median. Box represents the 25% and 75% quartile. Asterix represents the mean. Circled points: Values outside the upper whisker (min(max(x), 75% quantile +1.5*IQR) and outside the lower whisker (max(min(x), 25% quantile+1.5*IQR)  =  outliers. IQR  =  interquartile range (75% quantile–25% quantile).

The body mass of the female mutant *Zscan10* cohort was slightly, but significantly, (p = 0.042) reduced (see Figure 1A-D); in male mutant mice a reduction was observed but did not reach a significant value. The relationship between body fat content and lean mass was not different between mutant and wild type mice, therefore no clear shift in body composition could be detected (see Figure 1D,E,G). In the dual-energy X-ray absorptiometry (DXA) a significantly decreased bone mineral content (BMC) and bone content was observed in female mutants (see Figure 1E). The tibia length was significantly reduced in female mice (see Figure 1F).

### Abnormalities of *Zscan10* homozygous mutant mice revealed by immunological, hematological and clinical chemistry screening

**Figure 2:**
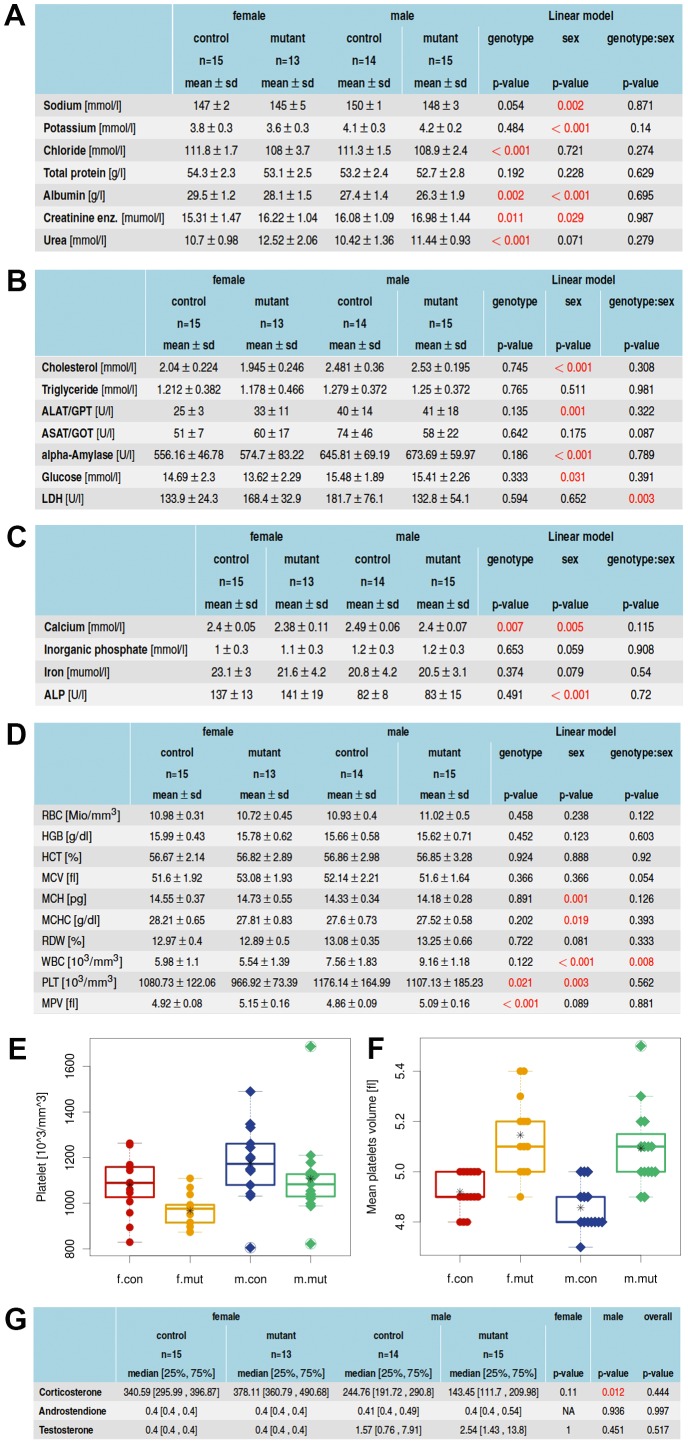
Hematological and clinical chemistry analysis. (A) Plasma electrolyte, protein, creatinine and urea concentrations of ad libitum fed mice. (B) Lipid and glucose levels as well as selected enzyme activities in plasma of ad libitum fed mice. (C) Plasma concentrations of minerals, iron and ALP activity of ad libitum fed mice. (D) Values obtained from EDTA-blood samples. Means, standard deviations and p-values for genotype, sex and genotype x sex interaction effects calculated by a linear model (A-D). Platelet (E) and mean platelet volume (F) boxplot strip chart, split by sex and genotype. (G) Concentration of steroid hormones in plasma: Medians, first and third quartile and p-values calculated by a Wilcoxon rank-sum test (n = 57). Points are individual animals; circles represent females (f); diamonds represent males (m) of *Zscan10* homozygous mutants (mut) and wild type littermates (con). Line within the boxplot represents median. Box represents the 25% and 75% quantile. Asterix represents the mean. Circled points: Values outside the upper whisker (min(max(x), 75% quantile +1.5*IQR) and outside the lower whisker (max(min(x), 25% quantile+1.5*IQR)  =  outliers. IQR  =  interquartile range (75% quantile–25% quantile).

The clinical-chemical and hematological screen detected several changes indicating effects of the *Zscan10* mutation on renal function as well as platelet number and morphology. The glucose metabolism and tolerance was unaffected in an intraperitoneal glucose tolerance test (IpGTT) (data not shown). *Zscan10* mutant mice presented with moderately decreased chloride and mildly reduced albumin concentration in plasma, while creatinine and urea levels were increased in these mice compared to controls (see Figure 2A). Plasma lactate dehydrogenase (LDH) activity generally present in body tissues, such as blood cells and heart muscle, is released upon tissue damage, and therefore a valuable indicator thereof. LDH activity was significantly increased in female mutant mice, while it tended to be lower in mutant males (see Figure 2B). Calcium was decreased in male mutants compared to male controls, while no effect was observed in the female cohorts (see Figure 2C). Alkaline phosphatase (ALP) activity was not increased in mutant animals (see Figure 2C). No significant effect on red blood cell count or morphology was observed, platelet counts were significantly decreased and the mean platelet volume (MPV) was increased in *Zscan10* mutants (see Figure 2D,E,F). A higher frequency of T-cells in mutants went together with a lower CD4^+^/CD8^+^ ratio. The frequency of CD25-expressing cells within the CD4^+^ T-cell cluster, the frequency of CD11b expressing cells within the NK-cell cluster, and the frequency of MHC class II-expressing cells within the B-cell cluster was higher in mutants, also higher levels of IgG1, IgG2b and an increased ratio of IgG1/IgG2b was detected in mutants. Plasma IgE levels were unaffected (see Figure S2). Concentrations of corticosterone, androstendione and testosterone in the plasma of *Zscan10* mutant and control mice aged 16 weeks revealed a statistically significant decrease in corticosterone levels in male mutants (see Figure 2G).

### Neurological and behavioral abnormalities in *Zscan10* homozygous mutant mice

**Figure 3:**
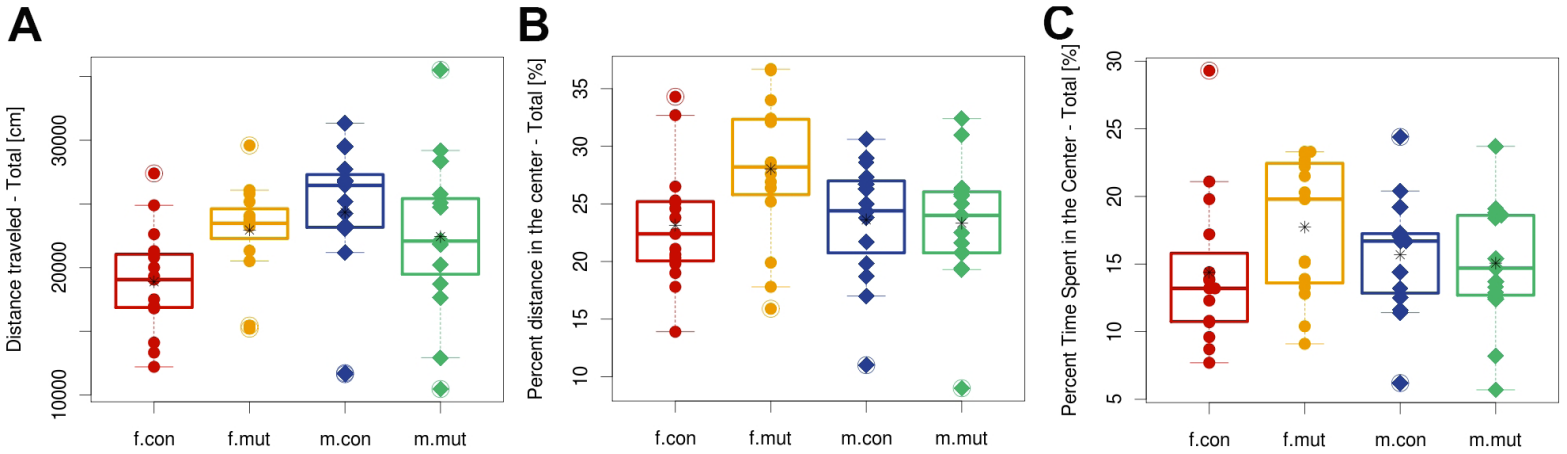
Open Field Testing. Distance traveled (A), percent distance in the center (B) and percent time spent in the center (C). Total boxplot with strip chart, split by sex and genotype. Points are individual animals; circles represent females (f); diamonds represent males (m) of *Zscan10* homozygous mutants (mut) and wild type littermates (con). Line within the boxplot represents median. Box represents the 25% and 75% quantile. Asterix represents the mean. Circled points: Values outside the upper whisker (min(max(x), 75% quantile +1.5*IQR) and outside the lower whisker (max(min(x), 25% quantile+1.5*IQR)  =  outliers. IQR  =  interquartile range (75% quantile–25% quantile).

Female mutant mice showed increased locomotor activity (total distance travelled) and increased locomotor speed (p<0.05) in the open field and entered the center more frequently while male mutant mice showed the opposite effect (interactions genotype x sex: p<0.05) (see Figure 3A,B,C and Figure S3). Male mutant mice also exhibited increased PPI and female mutants exhibited an increase in acoustic startle reactivity at higher sound pressure levels (data not shown).

SHIRPA is a standardized observation protocol for assessment of genetically modified mice. During this test increased occurrence of urination was detected for two thirds of the mutant cohort, compared to only one third of the wild type mice (p = 0.00921, n = 30). Measuring grip strength for the evaluation of muscle function revealed reduced force in 2- and 4-paw measurement. However, the mutant mice were also significantly lighter (see Figure 1A,B,C) and including body mass as a confounding factor into statistical analysis confirmed body mass as the main effect on grip strength. During Rota rod analysis, mutants fell off the Rota rod more often while controls rotated passively, a behavior also rated as a failure thus leading to manual timer stop. This difference in the strategy on the rod, however, had no influence on mean latencies (data not shown). Other parameters covering overall appearance, movement and reflexes were without any significance.

### Reduced eye size in *Zscan10* homozygous mutant mice

**Figure 4:**
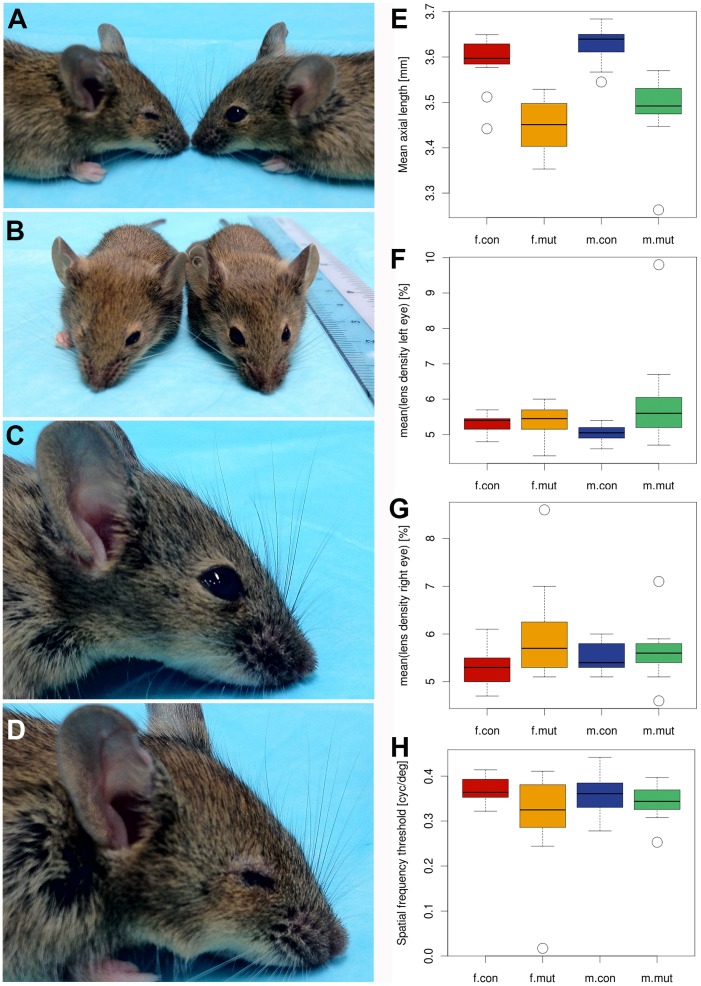
Small eye phenotype in *Zscan10* homozygous mutant mice. Lateral (A) and frontal (B) view of adult mutant (left) and wild type (right) mice. Lateral view of right eye of a wild type (C) and mutant (D) adult female. (E) Eye size measurements by laser interference biometry revealed significantly reduced axial eye length in mutants of both sexes (p<0.001). Anterior eye investigation by Scheimpflug imaging indicated significantly increased mean lens density between controls and mutants for left eyes in males (p = 0.002) (F) and right eyes in females (p = 0.016) (G). Virtual drum vision testing showed a reduced response with borderline significance in female mutants (p = 0.034) (H). (E) to (H) are displayed as boxplot split by sex and genotype. Outliers are indicated by open circles.

Significantly reduced eye sizes in both male and female *Zscan10* mutants were detected, present already at midgestation (see Figure 4A-D and data not shown). Laser interference biometry revealed significantly reduced axial eye lengths in mutants of both sexes (P<0.0001) (see Figure 4E). Borderline significant increased opacification of left and right lenses of male and female mutants, respectively, was observed. Scheimpflug imaging indicated significantly increased mean lens density between controls and mutants, yet with a surprising unilateral restriction to left eyes in mutant males (p = 0.002) and right eyes in mutant females (p = 0.016) (see Figure 4F,G). Optical coherence tomography demonstrated a normally developed fundus with no significant alteration in the number of main blood vessels or retinal thickness in the posterior part of the eye. While male mutants showed regular responses to the moving stripe pattern in the virtual drum vision test, female mutants showed a reduced response with a borderline significance (p = 0.034) (see Figure 4H).

### Further phenotypic findings in *Zscan10* homozygous mutant mice

Awake echocardiography of *Zscan10* homozygous mutants and respective controls revealed increased systolic left ventricular internal dimensions (LVIDs) and a decreased respiration rate mainly in male mutants. Further, *Zscan10* mutants showed a decreased heart rate when compared with control mice (data not shown). Thus, only mild genotype-related differences without relevance for the cardiovascular system were found. Lung function screening showed a slight yet significant but functionally rather irrelevant decreased dynamic compliance (p = 0.026) and tidal volume (p = 0.004) (data not shown). Body temperature showed a tendency to be slightly lower in male mutants (data not shown).

**Figure 5:**
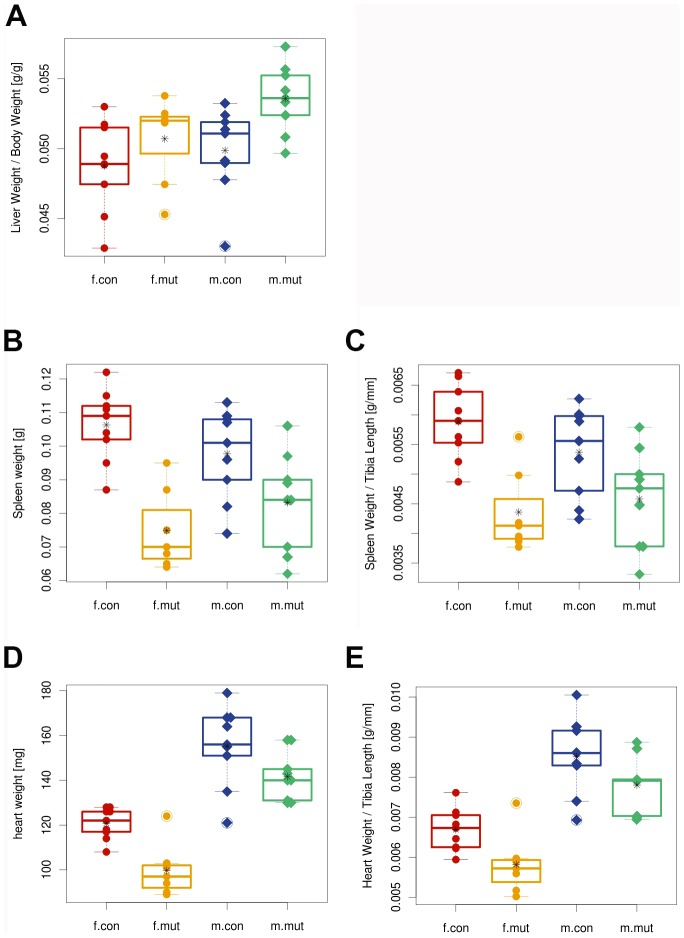
Organ weight determined in pathology screen. (A) Normalized liver weight is significantly increased in male mutant mice (P = 0.008). (B) Absolute spleen weight is significantly reduced in mutant mice of both genders. (C) Normalized spleen weight is significantly reduced in female mutant mice (P = 0.002). (D) Absolute heart weight is significantly reduced in female mutant mice (p = 0.003). (E) Normalized heart weight is significantly reduced in female mutant mice (p = 0.016). All results demonstrated as boxplot with strip chart, split by sex and genotype. For tibia length refer to Figure 1F. Points are individual animals; circles represent females (f); diamonds represent males (m) of *Zscan10* homozygous mutants (mut) and wild type littermates (con). Line within the boxplot represents median. Box represents the 25% and 75% quantile. Asterix represents the mean. Circled points: Values outside the upper whisker (min(max(x), 75% quantile +1.5*IQR) and outside the lower whisker (max(min(x), 25% quantile+1.5*IQR)  =  outliers. IQR  =  interquartile range (75% quantile–25% quantile).

In homozygous male mutant mice an increase in liver weight normalized to body weight was determined (see Figure 5A). A slightly reduced absolute and normalized spleen and heart weight in particular in female mutants was observed (see Figure 5B-E, and Figure S4), yet no morphological changes of internal organs were detected during histological analysis.

### *Zscan10* expression in midgestation embryos supports phenotypic findings

**Figure 6:**
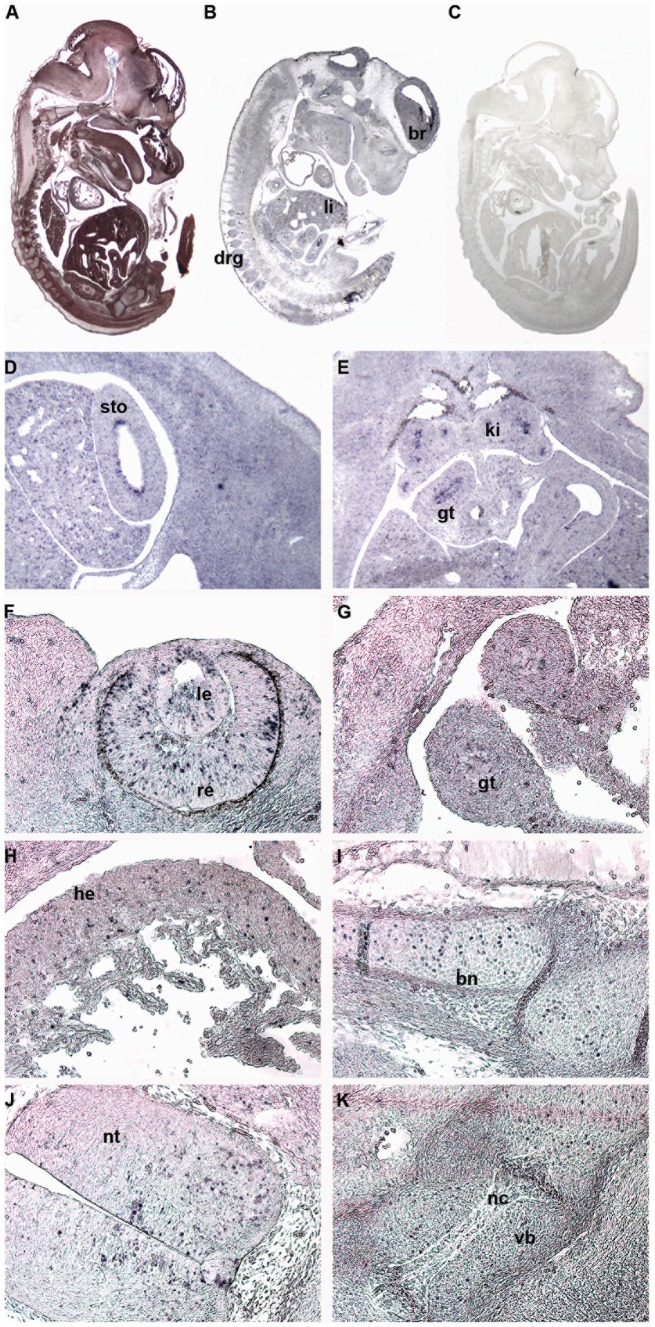
Punctuated *Zscan10* expression in E12.5 wild type embryos as determined by SISH. Sagittal sections through E12.5 wild type embryos for reference as Mallory tetrachrome staining (A), with T7 antisense probe (B) and T3 sense probe (C). T7 antisense probe on transverse sections reflecting *Zscan10* expression in the lining of the stomach (D), kidney and lining of the gut (E) at 4x; on sagittal sections through the eye (F), lower abdomen (G), heart (H), humerus (I) and vertebral body (K), as well as transverse sections through the neural tube (J) at 10x. All pictures were taken on a Motic compound microscope with a Motic Moticam 2.0MP digital camera. Brain (br), liver (li), dorsal root ganglia (drg), stomach (sto), kidney (ki), gut (gt), lens (le), retina (re), heart (he), bone (bn), neutral tube (nt), notochord (nc) and vertebral body (vb).

**Figure 7:**
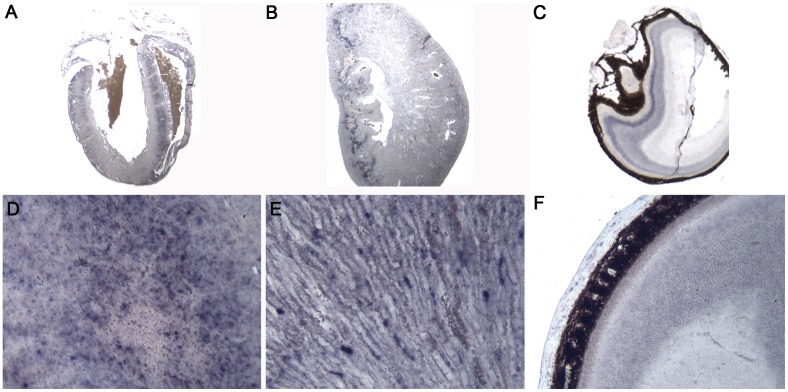
Punctuated *Zscan10* expression persists in adult tissue. SISH on sections through heart (A,D), kidney (B,E) and eye (C,F) probed with the T7 *Zscan10* antisense probe taken at 0.63× (A,B) and 2.5× (C) on a Leica S6 dissecting scope and magnifications thereof (D,E,F) taken on a Motic compound microscope at 10× all documented with a Motic Moticam 2.0MP digital camera.

Phenotypically affected organs in the screen performed by GMC led us to revisit embryonic and adult *Zscan10* expression. Since expression pattern analysis was previously established by WISH [3], [6], a technique prone to probe entrapment, lack of proper penetration of the probe and other obstacles, SISH was performed. A 270 bp probe essentially spanning exons 3 to 5 and a 1.35 kb probe essentially covering exon 2 to the proximal part of exon 6 was used and compared with an EGFP probe reflecting the pUPA reporter gene expression (see Figures S1 and S6). As determined by repeat masker (www.repeatmasker.org), the *Zscan10* probes were free of repetitive elements and specific to *Zscan10*. In several independent experiments (n = 6) we observed a punctate expression pattern (a positive cell surrounded by numerous non-expressing cells) in E12.5 and E15.5 embryos, namely in kidneys, heart, liver, muscle and developing bones of the forelimb, but in a similar matter in neural tissue like the neural tube, the brain and in dorsal root ganglia and sensory organs like the eye (see Figure 6B,D-K). Strongest expression of such kind was located in the linings of stomach and gut (see Figure 6D,E,G). This punctate expression pattern for *Zscan10* was still observed in the organs of adult mice as shown for sections of the heart, kidney and eye (see Figure 7). Expression of the pUPA reporter gene EGFP coincided with *Zscan10* expression (see Figure S6M-X). The bHLH transcription factor *Hes5* [27], a downstream effector in the Notch pathway [28] involved in eye, especially retinal development [29], appeared reduced in its signal strength in the *Zscan10* ^−/−^ mutant retina versus that of a heterozygous littermate, yet this would require further quantitative analysis (see Figure S6C,I). No change in expression for *Hes5* was observed in other organs analyzed. *Sox2* was found expressed in the heart, neural tube and eye similar to, but at much lower levels than *Zscan10*, equivalent expression was observed in the apical ectodermal ridge. No coexpression was detected in the vertebrate body and for the homozygous mutant in kidney and adrenal (see Figure S6Y-P′).

## Discussion

*Zscan10* was originally discovered in screens of human and mouse ESC to identify genes with relevance in maintaining a pluripotent state of these cells. The established interaction of Zscan10 with known pluripotency markers like Oct4 and Sox2 in ESC suggested a potentially crucial role of *Zscan10* in early mouse development [1]. Oct4 is a homeodomain transcription factor of the POU family, highly expressed in ESC and with a critical function in maintaining pluripotency [30]. In its absence an ICM does not form [31]. *Oct4* becomes silenced upon differentiation with the exception of its requirement for the viability of germ cells [32]. Ectopic expression of *Oct4* from the ROSA26 locus resulted in dysplasia in the gastrointestinal tract and skin due to an increase in progenitor cells [33]. Oct4 is capable of heterodimerization with Sox2, a transcription factor and member of the SRY-related HMG-box (Sox) family. Heterozygous mutations in the *Sox2* gene have been linked to eye malformations like anophthalmia and microphthalmia [34], [35]. Sox2 is further known to interact with Pax6, a master control gene during eye development [36], [37], [38] with pleiotropic effects in mouse and human when mutated amongst some are the proliferative control of neural stem or progenitor cell populations [39] and pancreatic α-cell development [40]. Notably the pancreas and spleen originate from a shared embryonic lineage [41].

### *Zscan10* mutants partially phenocopy Sox2 and Pax6 hypomorphs

We have successfully generated fertile homozygous mutant mice for *Zscan10* using the gene trap clone #285B6 obtained from CMMR. Our initial phenotype assessment indicated a slight decrease in the number of homozygous mutant females at weaning age as well as a size reduction in homozygous mutant mice. A more detailed phenotype analysis conducted by the GMC confirmed significantly reduced body weight, a significant reduction in absolute heart weight, tibia length, and a reduced bone mineral content was observed in homozygous female mutants, however ALP activity was not increased in mutant animals as usually seen in mice with increased bone turnover. Furthermore a significant reduction in absolute spleen weight in both genders was noted, while the liver weight was only significantly increased in homozygous males.

A detailed eye screen conducted by GMC identified a significant reduction in the eye size and axial eye length in both genders. There was also a borderline significantly increased opacification, yet no abnormalities in retinal layers or vascularization of the fundus of homozygous mutants. Overall, this resulted in reduced vision in the female mutants. Aniridia, anophthalmia and microphthalmia have been associated with haploinsufficiency of SOX2 while anterior eye defects, including defects of the iris and ciliary body have been associated with haploinsufficiency of PAX6 in humans [34], [37], [42], [43], [44], [45], [46], [47]. Analysis of eye development in *Sox2* hypomorphic mice indicated variable degrees of microphthalmia attributed to abnormal neural progenitor proliferation, consistent with findings in human [48]. A follow up study with conditional *Sox2* mutant mice revealed an inversely correlated gradient between *Sox2* and *Pax6* expression in the developing optic cup and demonstrated the importance of the functional antagonism between Sox2 and Pax6 during patterning of the eye cup [49]. Hes5 is a known downstream effector in the Notch pathway, important in the cell fate decision of retinal progenitor cell populations [29]. We observed a slightly reduced expression of *Hes5* in Zscan10 mutant retinas. The previously demonstrated physical *in vitro* interaction between Zscan10 and Sox2 [1], the coexpression of *Zscan10* and *Sox2* demonstrated here in the eye and the small eye phenotype identified during the GMC phenotyping screen nominates Zscan10 as a new player in the concerted interaction of transcription factors during eye development.

### Altered platelet counts and an activated immune status in *Zscan10* homozygous mutants

The finding, that a slightly decreased platelet count is associated with an increase mean platelet volume (MPV) in *Zscan10* mutant mice compared to controls, fits to the observation that these parameters are usually negatively correlated [50], [51]. Increased MPV values and the occurrence of “large platelets” can indicate changes in cytoskeletal structures or an increased proportion of pre-platelets and pro-platelets (prePLT, proPLT) as well as young platelets, which can occur in response to thrombopenia [51]. Increased platelet size goes together with higher platelet reactivity and is associated with diabetes mellitus and an increased risk of cardiovascular diseases in humans and is therefore considered as an important diagnostic marker [50], [52], [53]. A recent GWAS study identified 68 loci associated with platelet number and volume [54]. The slightly decreased platelet count in homozygous *Zscan10* mutants associated with increased MPV values could therefore result from changes in number and function of megakaryocytes – which could be in line with the suggested role of Zscan10 in maintaining sufficient numbers of progenitor cells - resulting in effects on cell differentiation, platelet formation or platelet maturation. A decrease in the number of megakaryocytes could be compensated by an increased activity of these cells leading to the observed differences, as it was described for the response to pharmacologically induced thrombopenia [50], [51]. On the other hand these changes could occur due to changes in cytoskeletal structure or secondarily to an increased platelet consumption or destruction. Further investigations are necessary to elucidate the underlying cause.

### *Zscan10* homozygous mutant mice display behavioral alterations

The Open Field Test was originally described to study emotionality in rats [55] with emotional rats executing fewer entries in the central part of the test arena and higher levels of defecation. Glucocorticoid hormone levels can be indicative of stress tolerance with corticosterone levels being elevated during periods of stress [56], [57]. The acoustic startle response and PPI measure auditory sensorimotor integration and an increased acoustic startle response can be indicative of increased anxiety levels. Impaired PPI has been associated with human disorders such as: schizophrenia, Huntington's disease, fragile X syndrome and autism and involves amongst other signaling centers the limbic cortex [58], [59]. The GMC steroid screen at 16 weeks of age indicated decreased corticosterone levels in plasma of *Zscan10* homozygous male mutant mice, and a trend in the opposite direction in female mutants. Significantly increased urination was observed in *Zscan10* mutants, female *Zscan10* mutants were slightly more active in the OFT, including more entries into and more distance travelled in the “anxiogenic” center zone of the OFT. It could be hypothesized based on the expression of *Zscan10* in the embryonic brain that an impact on a pluripotent progenitor cell population for example in the limbic system could influence anxiety related behavior along the way of observations made in *Dlx*1 loss-of function mutants [60], where the *Dlx1* sense [60], [61], [62], [63] and antisense transcript level is critical in progenitor fate decisions [15]. However, female *Zscan10* homozygous mutants also show an increased acoustic startle response, which is not consistent with the notion of reduced anxiety, and demonstrated a functional visual impairment in the optokinetic drum test. It cannot be excluded that this visual impairment might have played a role in the observed OFT behavior of female *Zscan10* homozygous mutants, since it could affect the perception of the arena and its center zone as anxiogenic. The increased urination observed in the neurology screen could however also be due to a renal dysfunction as indicated by the results from the clinical chemistry screen which revealed increased urea and creatinine levels as well was a slight hypocalcemia in males that among other causes can also be attributed to renal dysfunction.

Taken together, further neurological examinations and immunohistochemistry would be required to identify the exact nature of *Zscan10* homozygous mutant behavior.

### *Zscan10* remains expressed in potential progenitor cells of diverse organs

All phenotypic findings were coherent with the embryonic and adult *Zcan10* gene expression patterns established by SISH. Previous WISH expression patterns in midgestation embryos could have misinterpreted the punctate expression pattern of *Zscan10* as background staining. We propose based on a more refined expression pattern by SISH, the known interaction partners of Zscan10 [1] and the pleiotropic phenotype observed in *Zscan10* mutants, that Zscan10 could play a role in regulating the availability of progenitor cells during mouse embryonic development and adulthood. In the case of *Zscan10* hypomorphic or loss-of-function mutations the number of progenitors might be reduced, either by a decrease in progenitor cell division or by premature terminal differentiation causing an overall reduction in cell mass of a variety of target tissues namely eye, heart, long bones and spleen. Identified grip strength, immunological and blood chemistry abnormalities could be downstream effects of the impact on progenitor cell populations in the affected organs. The pluripotency marker *Sox2* is coexpressed in many but not all of these organs, yet generally in fewer cells compared to *Zscan10*. This is not surprising as progenitor cells might have acquired a certain maturity and as such are no longer of pluripotent nature, yet can act as multipotent progenitors for cells at an advanced differentiation level. The exact mechanism by which Zscan10 acts in a given progenitor cell population *in vivo* remains to be determined.

In summary, the observed eye phenotype in homozygous *Zscan10* mutant mice supports previously proposed *in vivo* interactions between Sox2 and Zscan10 based on earlier *in vitro* assays. The nature of the cellular expression pattern of *Zscan10* in midgestation embryos and adult organs and the changes in absolute weight of several organs as well as the composition of several cell populations in the homozygous mutants suggests a role for Zscan10 in maintaining a multipotent progenitor cell population or impacting on fate choice decisions thereof.

## Supporting Information

Figure S1 **Gene trap strategy.** Illustrated is the pUPA-TRAP vector (not drawn to scale) as used by CMMR that was integrated into intron 1 upstream of the ATG start codon. Exons of the currently known five *Zscan10* transcripts are drawn to scale as rectangular boxes, yellow and white representing coding and untranslated regions, respectively. The 270 bp SISH probe (purple lines) is covering most of exon 3, all of exon 4 and part of exon 5 and is drawn to scale for transcript 1. Transcripts after insertion of the pUPA-TRAP vector before and after Cre-recombination are indicated. Generation of Zscan10 protein after Cre-recombination is highly unlikely based on the design of the pUPA-TRAP vector. Transcription start site (TSS, black bend arrow), translation start (green arrow heads), translation stop (red arrow heads), splice acceptor site (SA), internal ribosome entry site (IRES), polyadenylation site (pA), RNA polymerase II promoter (RNAPII), recognition site for Cre recombinase (loxP), splice donor site (SD). Integration site of pUPA-TRAP vector is indicated by the blue arrow.
(EPS)

Figure S2 **Results of the immunological screen.** (A) Frequencies of leukocyte subpopulations during flow cytometry in peripheral blood after erythrocyte lysis [percentage of all leukocytes (CD45+ cells), or corresponding parent gate, respectively]. Means, standard deviation and p-values calculated by a linear model. (B) Levels of immunoglobulins (ug/ml) in blood plasma. Medians, first and third quartile and p-values calculated by Wilcoxon rank-sum test. Missing measurements caused by Ig levels above and below measurability were replaced by 0.9*min/1.1*max of respective Ig measurement (n = 57). (C) Total IgE in plasma were in normal range. Medians, first and third quartile and p-values calculated by a Wilcoxon rank-sum test.
(TIF)

Figure S3 **Open Field Testing.** Means, standard deviation and p-values calculated by a linear model (n = 60).
(TIF)

Figure S4 **Organ weight.** Medians, first and third quartile and p-values calculated by a Wilcoxon rank-sum test (n = 57).
(TIF)

Figure S5 **Western blot of the homozygous** ***Zscan10*** **mutant (−/−) ESC line L2 in comparison to wild type (+/+) and heterozygous (+/−) ESC.** Using 15 ug each with the GIS206-Ab [1], maintained on mouse embryonic fibroblasts (MEF) or on MEF-free gelatinized plates (GEL) and aRB-HRP as loading control.
(EPS)

Figure S6 ***Sox2*** **,** ***Hes5*** **and EGFP expression in comparison to** ***Zscan10*** **.** Sections through E15.5 (A-X) and E12.5 (Y-P′) *Zscan10*−/− (G-R, K′-P′) and *Zscan10*+/− (A-F, S-J′) mouse embryos showing expression of the transcription factors *Hes5* (A-L), *Zscan10* (S-D′) and *Sox2* (E′-P′) as well as the pUPA reporter EGFP (M-R). Organs expressing the Notch pathway effector *Hes5* did not show any obvious difference in *Hes5* expression between *Zscan10*−/− and *Zscan10*+/− embryos as shown for the brain (A,G) neural tube (nt) (B,H) gut (D,J) and liver (E,K) with exception of the retinal layer (C,I), where the *Hes5* signal appeared less intense, either due to reduced expression levels or fewer cells expressing *Hes5* in the *Zscan10^−/−^* eye (I). Ectopic upregulation of *Hes5* in chondrogenic tissue was not observed (F,L). EGFP expression was similar to *Zscan10* expression in all organs assayed for namely: Adrenal (M,S), kidney (N,T), heart (O,U), lung (P,V) vertebrae body and forming intervertebral disc (Q,W) and dorsal root ganglia (drg) (R,X). *Sox2* was not detected in kidney and adrenal (E′,K′), only faintly expressed in nt and drg (G′,M′) but found coexpressed with *Zscan10* in the heart (Z,F′,L′), the apical ectodermal ridge (aer) (C′,I′,O′) and the retina (D′,J′,P′).
(TIF)

Table S1 **Genotyping results from offspring at weaning age (n = 153).** P-value is calculated as one tailed probability values with n = 2 degrees of freedom.
(EPS)
