# Correction: Pleiotropic Functions for Transcription Factor Zscan10

**DOI:** 10.1371/journal.pone.0118766

**Published:** 2015-03-05

**Authors:** The PLOS ONE Staff

There are errors in the Competing Interests section. The correct competing interests information is as follows: This study was supported in part by the Agency for Science Technology and Research (A*STAR) Singapore. This does not alter our adherence to PLOS ONE policies on sharing data and materials.
