# Supplementary figures and images for: Pleiotropic Functions for Transcription Factor Zscan10
Source: PLoS One. 2014 Aug 11;9(8):e104568. doi: 10.1371/journal.pone.0104568 (PMC4128777; doi:10.1371/journal.pone.0104568)

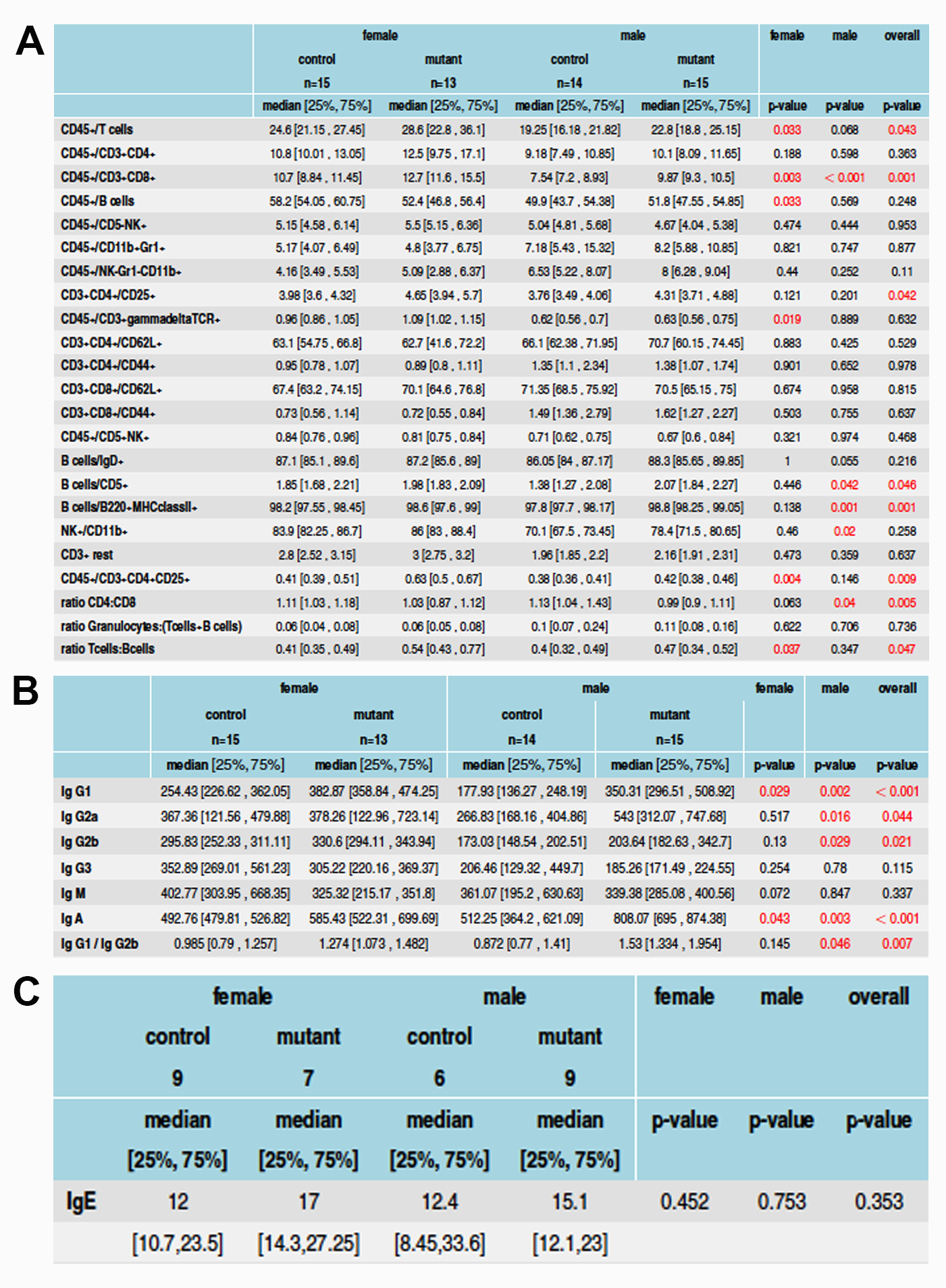

Supplement: Figure S2 — Results of the immunological screen. (A) Frequencies of leukocyte subpopulations during flow cytometry in peripheral blood after erythrocyte lysis [percentage of all leukocytes (CD45+ cells), or corresponding parent gate, respectively]. Means, standard deviation and p-values calculated by a linear model. (B) Levels of immunoglobulins (ug/ml) in blood plasma. Medians, first and third quartile and p-values calculated by Wilcoxon rank-sum test. Missing measurements caused by Ig levels above and below measurability were replaced by 0.9*min/1.1*max of respective Ig measurement (n = 57). (C) Total IgE in plasma were in normal range. Medians, first and third quartile and p-values calculated by a Wilcoxon rank-sum test. (TIF) [file pone.0104568.s002.tif]

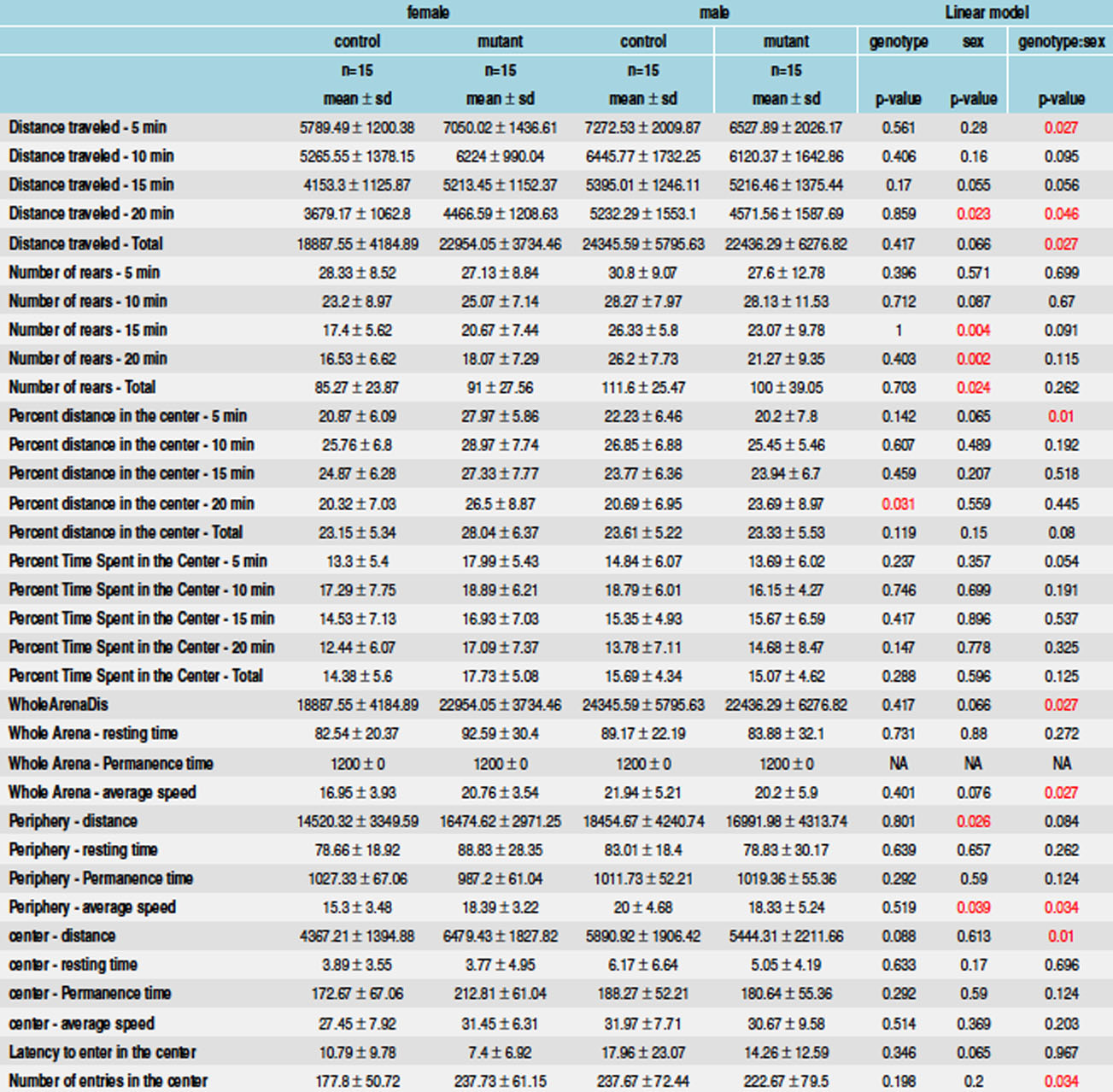

Supplement: Figure S3 — Open Field Testing. Means, standard deviation and p-values calculated by a linear model (n = 60). (TIF) [file pone.0104568.s003.tif]

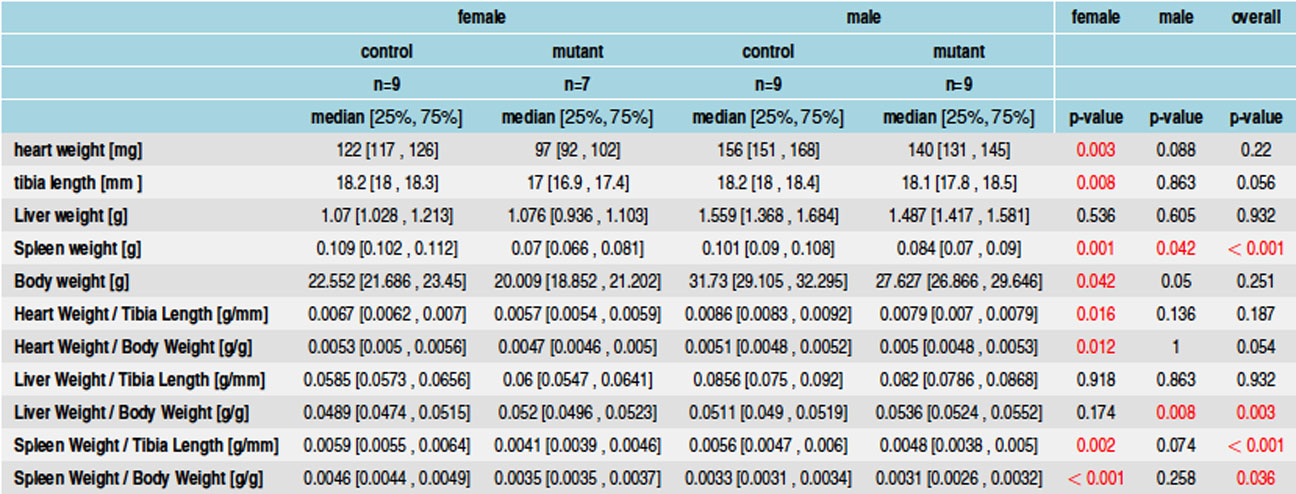

Supplement: Figure S4 — Organ weight. Medians, first and third quartile and p-values calculated by a Wilcoxon rank-sum test (n = 57). (TIF) [file pone.0104568.s004.tif]

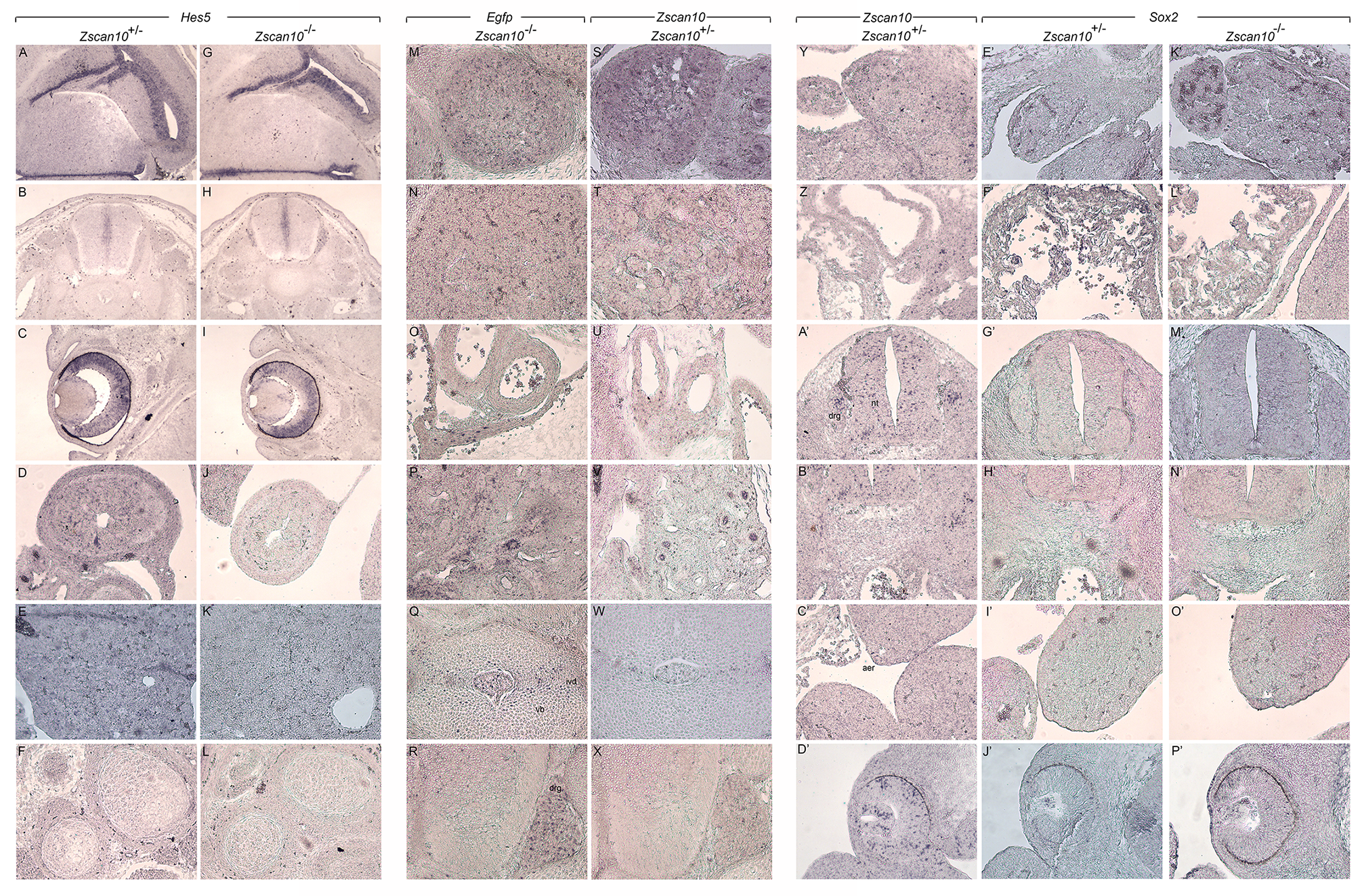

Supplement: Figure S6 — Sox2 , Hes5 and EGFP expression in comparison to Zscan10 . Sections through E15.5 (A-X) and E12.5 (Y-P′) Zscan10−/− (G-R, K′-P′) and Zscan10+/− (A-F, S-J′) mouse embryos showing expression of the transcription factors Hes5 (A-L), Zscan10 (S-D′) and Sox2 (E′-P′) as well as the pUPA reporter EGFP (M-R). Organs expressing the Notch pathway effector Hes5 did not show any obvious difference in Hes5 expression between Zscan10−/− and Zscan10+/− embryos as shown for the brain (A,G) neural tube (nt) (B,H) gut (D,J) and liver (E,K) with exception of the retinal layer (C,I), where the Hes5 signal appeared less intense, either due to reduced expression levels or fewer cells expressing Hes5 in the Zscan10−/− eye (I). Ectopic upregulation of Hes5 in chondrogenic tissue was not observed (F,L). EGFP expression was similar to Zscan10 expression in all organs assayed for namely: Adrenal (M,S), kidney (N,T), heart (O,U), lung (P,V) vertebrae body and forming intervertebral disc (Q,W) and dorsal root ganglia (drg) (R,X). Sox2 was not detected in kidney and adrenal (E′,K′), only faintly expressed in nt and drg (G′,M′) but found coexpressed with Zscan10 in the heart (Z,F′,L′), the apical ectodermal ridge (aer) (C′,I′,O′) and the retina (D′,J′,P′). (TIF) [file pone.0104568.s006.tif]
